# Supplementary material for: Consumption of Ultra-Processed Food and Drinks and Chronic Lymphocytic Leukemia in the MCC-Spain Study
Source: Int J Environ Res Public Health. 2021 May 20;18(10):5457. doi: 10.3390/ijerph18105457 (PMC8160774; doi:10.3390/ijerph18105457)
Supplement: Supplementary file 1 [file ijerph-18-05457-s001.zip › ijerph-1201660-supplementary.pdf]

<u>Supplementary material</u>

Consumption of ultra-processed food and drinks and chronic lymphocytic leukemia in the MCC-Spain study

Flow Chart

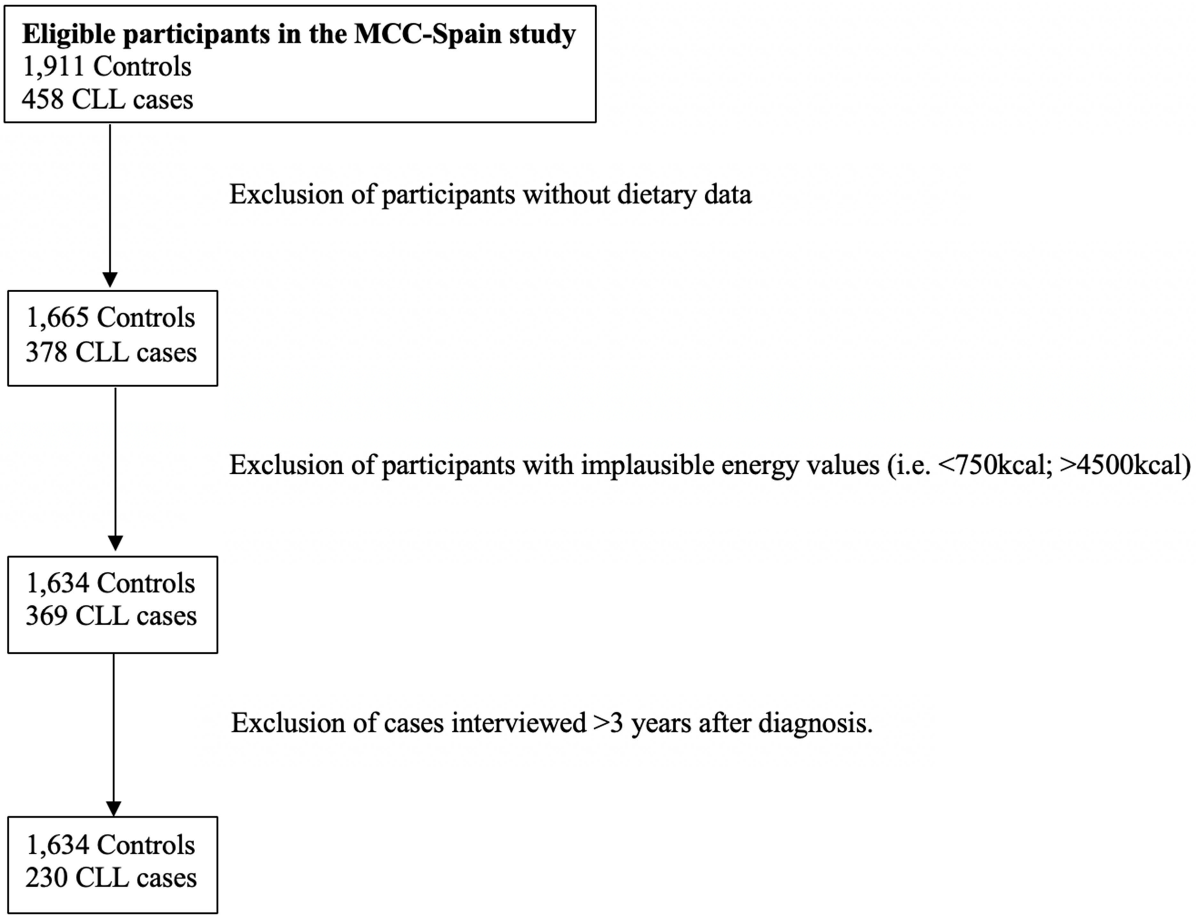

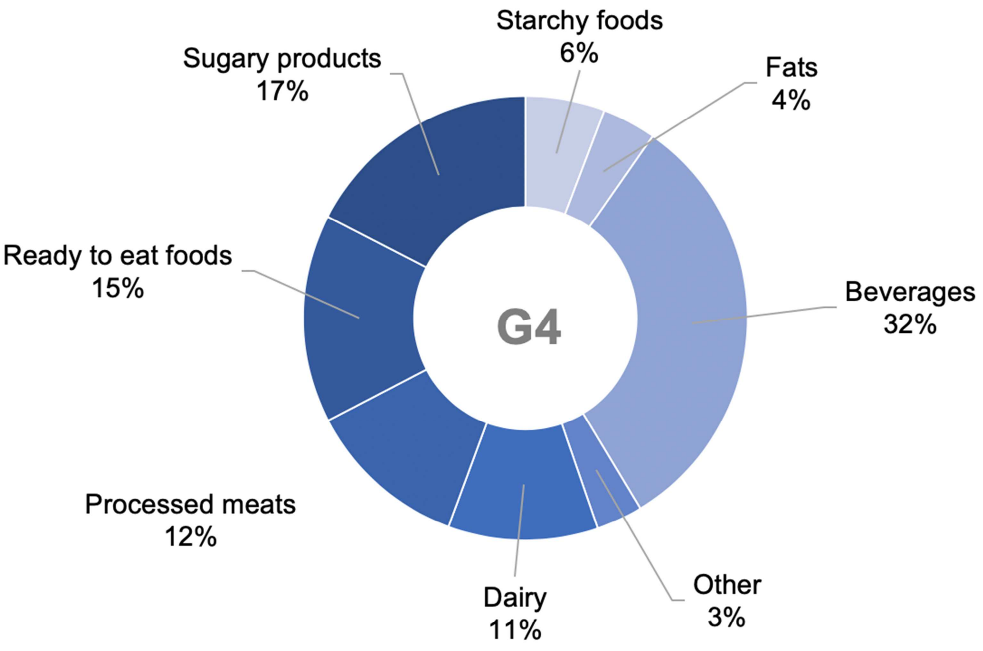

Figure S1. Relative contribution of each food group to ultra-processed food and drinks consumption (NOVA classification, group 4) in controls of the MCC-Spain study.

Table S1. Classification of foods according to the NOVA definition in the MCC-Spain study.

**G4:** Ultra-processed foods and drinks	Processed meats[a] (sausages, hamburgers, meat balls, cold meats, hot dog, pâté), croquettes, crab sticks, pastries, kebab, soya milk, custard, milkshakes, ice creams, breakfast cereals, wholegrain breakfast cereals, breadsticks, ravioli, pizza, instant soup, mayonnaise (normal and light), tomato sauce[b], cream, other sauces, margarine, cookies, pastries, churros, chocolate, chocolate powder, turrón (nougat), mantecado, chips, spirits, sugar-sweetened beverages, non-sugar sweetened beverages, packed fruit juices, tomato juice, tiger nut milk, soda water, saccharin and sweeteners.
**G3:** Processed foods	Bacon, ham, canned fish and seafood, cured fish, canned fruits, canned vegetables, fruits in syrup, olives, dry fruits, condensed milk, cheeses, white bread, whole grain bread, jam, wine, beers and champagne.
**G2:** Processed culinary ingredients	Salt, olive oil, sunflower oil, butter, lard and sugar.
**G1:** Unprocessed or minimum processed foods	Whole milk and skimmed milk, whole and skimmed yogurts[c], meat (chicken, turkey, pork, beef, lamb, rabbit), liver, offal, fish, seafood, fresh vegetables, homemade gazpacho and vegetable puree[b], legumes, fresh fruits, nuts[d], potatoes, rice, pasta, natural fruit juices, sparkling water and coffee.

A working group was created, including dietitians-nutritionists and experts in nutritional epidemiology, to classify foods and drinks into the NOVA groups. For some items, not enough information regarding food processing was available in the FFQ to unequivocally classify them into one of the four NOVA categories. In such cases, a consensus was reached based on the food composition table used, the labels of the products, and the information of usual consumption from external sources [1,2]:

[a]Although some burgers and meatballs can be artisanal, we assumed that most of them would be industrial (i.e. they contain added ingredients such as sulphites and flavours).

[b]Based on the household consumption data base of fruits and transformed vegetables [3], tomato sauce was classified as ultra-processed food (G4), since it is typically consumed in the form of industrial fried tomato rather than plain tomato sauce. On the other hand, recipes based on vegetables (i.e. gazpacho and vegetable puree) tend to be mostly homemade and thus, were classified as minimally process foods (G1).

[c]In the MCC FFQ, we could not distinguish between plain yogurt and sweetened and flavored yogurt. We considered yogurt to be unprocessed or minimum processed foods (G1).

[d]Several types of nuts can have a level of processing but, according to the Spanish household consumption database [4], raw walnuts are the most commonly consumed nuts.

References:

1. Palma I Cantós D FA. Tablas de Composición de Alimentos Por Medidas Caseras de Consumo Habitual En España. CESNID. Madrid: McGraw-Hill Interamerican; 2008.
2. Ministerio de Agricultura Pesca y Alimentación. [Base de datos de consumo en hogares] - Alimentación - magrama.gob.es. www.mapa.gob.es/app/consumo-en-hogares/consulta11.asp. Accessed August 6, 2019.
3. Gobierno de España. Ministerio de Agricultura y pesca alimentación y medio ambiente. Base de datos de consumo en hogares. Consumo de frutas y hortalizas transformadas. [Internet] [cited 2020 Feb 18]. Available from: http://www.mapama.gob.es/app/consumo-en-hogares
4. Gobierno de España. Ministerio de Agricultura y pesca alimentación y medio ambiente. Base de datos de consumo en hogares. Consumo frutos secos. [Internet] [cited 2020 Feb 18]. Available from: http://www.mapama.gob.es/app/consumo-en-hogares

Table S2: Characteristics of cases according to consumption of ultra-processed foods (NOVA classification, group 4) in the MCC-Spain study.

Only cases (n=230)	Ultra-processed food and drinks consumption (G4)[1]			
	Low (n=76)	Medium (n=75)	High (n=79)	P-value[2]
**Proportion of UPF consumption [(g/ g total intake) *100], mean (SD)**	5.0 (1.8)	11.2 (2.5)	26.6 (11.8)	**<0.001**
**Province, n(%)**				0.303
Barcelona	44 (57.9)	51 (68.0)	53 (67.1)	
Asturias	16 (21.1)	6 (8.0)	7 (8.9)	
Cantabria	2 (2.6)	3 (4.0)	2 (2.5)	
Granada	8 (10.5)	8 (10.7)	6 (7.6)	
Girona	6 (7.9)	7 (9.3)	11 (13.9)	
**Age (years), mean (SD)**	67.9 (8.3)	66.3 (10.7)	62.9 (10.6)	**0.007**
**Sex, n(%)**				0.521
Male	48 (63.2)	52 (69.3)	48 (60.8)	
Female	28 (36.8)	23 (30.7)	31 (39.2)	
**Education, n(%)**				**0.010**
Primary	55 (72.4)	37 (49.3)	37 (46.8)	
Secondary	14 (18.4)	21 (28.0)	27 (34.2)	
University	7 (9.2)	17 (22.7)	15 (19.0)	
**Body mass index[3] (kg/m^2), mean (SD)**	26.7 (3.9)	27.2 (4.3)	28.0 (4.3)	0.136
**Energy intake (kcal/day), mean (SD)**	1763.9 (524.9)	1953.2 (573.0)	2191.1 (667.3)	**<0.001**
**Alcohol consumption, median (IQR)**	3.5 (0.0-10.5)	6.0 (1.1-11.5)	5.1 (0.4-12.6)	0.991
**Smoking status, n(%)**				0.229
Never	27 (35.5)	29 (38.7)	37 (46.8)	
Former	10 (13.2)	12 (16.0)	16 (20.3)	
Current	39 (51.3)	34 (45.3)	25 (31.6)	
Unknown			1 (1.3)	
**Physical activity[4], n(%)**				0.972
Inactive	30 (39.5)	28 (37.3)	29 (36.7)	
Moderately active	10 (13.2)	10 (13.3)	12 (15.2)	
Active	9 (11.8)	8 (10.7)	12 (15.2)	
Very active	27 (35.5)	28 (37.3)	25 (31.6)	
Unknown		1 (1.3)	1 (1.3)	
**Ever worked in farming or agriculture, n(%)**				**0.011**
No	40 (52.6)	50 (66.7)	61 (77.2)	
Yes	36 (47.4)	25 (33.3)	17 (21.5)	
Unknown			1 (1.3)	
**Family history of hematological malignancy, n(%)**				0.652
No	65 (85.5)	63 (84)	65 (82.3)	
Yes	6 (7.9)	10 (13.3)	10 (12.7)	
Unknown	5 (6.6)	2 (2.7)	4 (5.1)	

Table S3: Association between ultra-processed food and drinks consumption (NOVA classification, group 4) and chronic lymphocytic leukemia, stratified by sex, age, smoking status, and alcohol consumption in the MCC-Spain

	**Total CLL cases**			**Incident CLL cases**		
	**N cases/ controls**	**OR per 10% increase[1] (95% CI)**	**P-int[3]**	**N cases/ controls**	**OR per 10% increase[1] (95% CI)**	**P-int[3]**
**By sex**			0.984			0.572
Women	76/638	**1.25 (1.00; 1.55)**		39/638	**1.49 (1.14; 1.95)**	
Men	146/928	1.00 (0.82; 1.20)		55/928	1.05 (0.79; 1.40)	
**By age**			0.839			0.505
<55	33/323	1.03 (0.73; 1.46)		18/323	1.50 (0.97; 2.31)	
55-74	143/953	1.13 (0.95; 1.35)		56/953	1.21 (0.94; 1.56)	
75	46/290	0.94 (0.64; 1.41)		20/290	1.08 (0.63; 1.86)	
**By smoking status**			0.055			0.414
No	87/662	**1.37 (1.11; 1.70)**		36/662	**1.49 (1.11; 1.99)**	
Former	97/617	0.81 (0.62; 1.05)		42/617	1.09 (0.78; 1.52)	
Current	38/285	1.07 (0.78; 1.46)		16/285	1.06 (0.68; 1.65)	
**By alcohol status[2]**			0.655			0.408
No consumption	51/276	1.06 (0.80; 1.40)		25/276	1.15 (0.80; 1.65)	
T1	64/418	1.08 (0.83; 1.40)		28/418	1.35 (0.98; 1.88)	
T2	60/442	1.08 (0.79; 1.48)		27/442	1.14 (0.71; 1.84)	
T3	47/430	1.04 (0.72; 1.48)		14/430	0.89 (0.47; 1.70)	

N, number; OR, odds ratio; CI, confidence interval; T: tertile

[1]Logistic regression model adjusted for age, sex, province, educational level, family history of hematological neoplasms, ever worked on farming, physical activity, energy intake, ethanol intake, and smoking status.

[2]Categories based on sex-specific distribution in controls.

[3]The p-value for interaction was calculated by modelling cross-product terms between UPF consumption (as continuous variables) and sex, age, smoking status, and alcohol intake.

In bold: p value <0.05
